# SLEEP HEALTH: IMPLICATIONS FOR CARDIOVASCULAR AND COGNITIVE FUNCTION

**DOI:** 10.1093/geroni/igae098.0849

**Published:** 2024-12-31

**Authors:** Kristen Knutson

**Affiliations:** Northwestern University Feinberg School of Medicine, Chicago, Illinois, United States

## Abstract

Healthy sleep is characterized by more than just adequate sleep duration or the absence of a sleep disorder. Indeed, there are numerous dimensions of sleep that are important for overall health, including sleep quality, sleep regularity and sleep timing. Sleep architecture is also an important dimension of sleep health. Prior research has demonstrated that sleep health is linked to numerous health domains, including cardiovascular and cognitive function. Further, sleep health varies by sociodemographic characteristics, such as age, gender and race, which has implications for disparities in both sleep health and in the consequences of poor sleep health, such as hypertension, cardiometabolic disease and cognitive impairment. This presentation will show data from two recent population-based studies that collected data on certain dimensions of sleep health. One study is from Brazil that collected polysomnography in people’s homes to assess sleep architecture and measured cardiovascular risk factors and cognitive function. The second study was conducted in Chicago, USA among adults 55 years of age and older and collected subjective and objective estimates of sleep and measured cardiovascular and cognitive function. This presentation will describe gender differences in the associations between sleep architecture and aging and in the associations between sleep architecture and CVD risk factors in Brazil. The racial differences in sleep health characteristics among older adults will be presented. Finally, associations between these various dimensions of sleep health and cognitive function from both studies will be described. Sleep health is multidimensional and is associated with health outcomes particularly important for older adults.

